# *FOXL2* drives the differentiation of supporting gonadal cells in early ovarian development

**DOI:** 10.1186/s12958-025-01377-0

**Published:** 2025-03-18

**Authors:** Laura Danti, Karolina Lundin, Petra Nedeczey-Ruzsák, Timo Tuuri, Juha S. Tapanainen

**Affiliations:** 1 Department of Obstetrics and Gynaecology, University of Helsinki and Helsinki University Hospital, P.O. Box 140, Helsinki, 00029 HUS Finland; 2 Department of Medicine Huddinge (MedH), Biosciences and Nutrition Unit, Karolinska Institutet, Huddinge, Sweden; 3 Department of Obstetrics and Gynaecology, HFR – Cantonal Hospital of Fribourg and University of Fribourg, Fribourg, Switzerland

**Keywords:** FOXL2, Ovary, Granulosa cell, CRISPR/Cas9, Stem cells

## Abstract

**Background:**

Forkhead box L2 (*FOXL2*) is a transcription factor from the forkhead box family primarily expressed in the pituitary, ovaries, and eyelids. Human mutations in *FOXL2* cause blepharophimosis, ptosis, epicanthus and inversus syndrome (BPES), which can be associated with primary ovarian insufficiency, and is indirectly linked with differences of sex development (DSD). Animal studies have shown the crucial role that *FOXL2* plays in the development, function, and maintenance of the ovary as well as in sex determination. However, the specific role of *FOXL2* in early human somatic cell ovarian development is largely unknown.

**Methods:**

In this study, we utilised CRISPR/Cas9 genome activation and a previously published in-house 14-day gonadal differentiation protocol to study the role of *FOXL2*.

**Results:**

Our results demonstrate that *FOXL2* downregulates coelomic epithelial markers *GATA4* and *LHX9*, female gonadal markers *RSPO1* and *WNT4*, and male gonadal markers *SOX9*, *NR0B1* and *DHH*. The differentially expressed genes were mostly associated with Kyoto encyclopaedia of genes and genomes (KEGG) pathways relating to cell adhesion molecules and gene ontology (GO) pathways relating to extracellular matrix and junction formation. Furthermore, a comparative analysis with existing single cell RNA sequencing data from human in vivo-derived samples elucidated that *FOXL2* initiates the downregulation of coelomic epithelial genes *GATA4*, *LHX9* and *UPK3B* at day 6. By day 8, the genes *ARX* and *GATA2* are transiently upregulated by *FOXL2* induction and then downregulated as the genes *LGR5*, *TSPAN8*, *OSR1* and *TAC1* become upregulated.

**Conclusions:**

These findings suggest that *FOXL2* facilitates the exit of differentiating cells from the coelomic epithelium and initially drives them towards a transitional identity before progressing into early supporting gonadal-like cells. The findings of this study significantly advance our understanding of normal gonadal development which can be used as a basis to elucidate pathological gonadal development underlying BPES.

**Supplementary Information:**

The online version contains supplementary material available at 10.1186/s12958-025-01377-0.

## Background

Human sex determination is a complex and delicate process, initially dictated by the configuration of sex chromosomes in an individual - XY in males and XX in females. Contrary to earlier beliefs suggesting that ovarian development is a default process, recent research shows it is an active mechanism that is initiated by the expression of specific genes whose combined role is to repress testicular development [1–3]. Key genes promoting female gonadal development are r-spondin 1 (*RSPO1*), wnt family member 4 (*WNT4*) and forkhead box L2 (*FOXL2*) [4]. Among these, *FOXL2* is considered one of the earliest markers of ovarian development [5, 6].

*The FOXL2* gene encodes a forkhead transcription factor that is primarily expressed in the ovary, pituitary gland, and eyelids. It plays a crucial role in mammalian sex determination and ovarian development, maintenance, and function [1, 2]. In humans, *FOXL2* mutations cause blepharophimosis, ptosis, epicanthus and inversus syndrome (BPES). This syndrome can be classified into two types dependent on the presence (type I) or absence (type II) of primary ovarian insufficiency (POI) [7].

The role of *FOXL2* in ovarian development has been extensively studied using animal models, the most notable being mice and goats. Homozygous knock-out (KO) mice exhibit a very high perinatal mortality rate and are characterised by small stature, along with eyelid and craniofacial abnormalities [8]. These mice are infertile due to abnormal follicle development, characterised by the presence of only a single layer of granulosa cells surrounding the growing oocyte, which fail to undergo the typical squamous to cuboidal transition. By eight weeks of age, the primordial follicle pool is already depleted, and widespread follicular atresia is observed. By sixteen weeks, no healthy oocytes or follicles remain [8, 9]. In goats, a naturally occurring *FOXL2* mutation causes polled intersex syndrome (PIS), which is associated with polledness and intersexuality. It solely affects XX individuals, resulting in female-to-male sex-reversal [10, 11]. In 2014, Boulanger et al. created a *FOXL2* goat KO model, where XX individual with bi-allelic *FOXL2* mutations exhibited complete sex-reversal and eyelid malformations, signifying that *FOXL2* is the female sex-determining gene in this species [12]. Additionally, Uhlenhaut et al. demonstrated in 2009 that *Foxl2* is required for ovarian maintenance in mice. Their study revealed that conditional deletion of *Foxl2* in mature granulosa cells of the postnatal ovary led to upregulation of the testis-determining gene *Sox9*. Consequently, the granulosa and theca cells of the ovary transdifferentiated into Sertoli cells and Leydig cells, respectively [3]. Together these KO studies solidified the crucial role for *FOXL2* in female sex-determination, as well as in ovarian development, function, and maintenance.

Despite these insights, the specific functional role of *FOXL2* during early ovarian development remains less well understood. In the present study, we employed CRISPR/Cas9 activation to conditionally induce *FOXL2* at the intermediate mesoderm stage in a 14-day gonadal differentiation protocol to investigate its function in early gonadal somatic cell development. Our results suggest that *FOXL2* acts as a trigger for the transition of cells from coelomic epithelium to an early supporting gonadal cell fate, marked by the expression of *LGR5* and *TSPAN8*. Furthermore, we demonstrate that *FOXL2* simultaneously represses the expression of genes that are known to drive male sex differentiation.

## Materials and methods

### Cell culture

H9-*FOXL2*-DDdCas9VP192 hESCs were cultured on Geltrex™ LDEV-Free Reduced Growth Factor Basement Membrane Matrix (Gibco, Thermo Fisher Scientific, Waltham, MA, USA) diluted in Dulbecco’s Modified Eagle Medium/Nutrient Mixture F-12 (DMEM/F-12) + Glutamax ^TM^ (Gibco, Thermo Fisher Scientific, Waltham, MA, USA). The cell culture plates were coated with Geltrex™ and cells were grown in Essential 8 ^TM^ (E8) medium (Gibco, Thermo Fisher Scientific, Waltham, MA, USA). When cells reached 70–80% confluency they were passaged in small colonies using Ultrapure™ ethylenediaminetetraacetic acid (EDTA, 0.5 mM) (Invitrogen, Thermo Fisher Scientific, Waltham, MA, USA). Cells were maintained in a CO_2_ incubator (PHCbi, Tokyo, Japan) set at 37 °C and 5% CO_2_.

H9-*FOXL2*-DDdCas9VP192 hESCs were differentiated following the gonadal differentiation protocol M described by Sepponen et al. [13]. Briefly, 12-well plates (Corning, Corning, NY, USA) were coated with human collagen I (0.5 μg/cm^2^) (Corning, Corning, NY, USA) dissolved in 2 mM HCl. Next, cells were dissociated from their culture plates into a single-cell solution using 0.5 mM EDTA and seeded at a density of 1.5 × 10^5^ cells/cm^2^ in 1 ml/well of day 0 medium onto the collagen-coated wells. Day 0 medium consisted of intermediate mesoderm (IM) medium (DMEM/F-12 + Glutamax™ + 2% B-27™ supplement (50X), serum-free (Gibco, Thermo Fisher Scientific, Waltham, MA, USA)) supplemented with 100 ng/ml activin A (ActA) (Q-kine, Cambridge, UK), 5 µM CHIR99021 HCl (CHIR) (Selleckchem, Cologne, Germany), 2 µM dorsomorphin 2HCl (DM) (Selleckchem, Cologne, Germany) and 10 µM rho kinase inhibitor Y27632 (ROCKi) (Selleckchem, Cologne, Germany). After 24h, the day 0 medium was replaced with 1 ml/well of day 1 medium, consisting of IM medium supplemented with 10 ng/ml bone morphogenic protein 7 (BMP7) (Peprotech, Cranbury, NJ, USA) and 3 µM CHIR. After another 24h, day 1 medium was removed, and day 2–3 medium (IM medium supplemented with 2 µM DM and 3 µM CHIR) was added to the cells at 2 ml/well for 48h. On day 4, after removing day 2–3 medium, control cells were cultured in 1.5 ml/well of IM medium and the induced cells were cultured in induction medium (IM medium supplemented with 1 µg/ml doxycycline hyclate (DOX; Sigma-Aldrich, St. Louis, MO, USA) and 1 µM trimethoprim (TMP; Sigma-Aldrich, St. Louis, MO, USA)). Cells were washed in between media changes with phosphate buffered saline (PBS, 1X) containing Magnesium and Calcium (Sigma-Aldrich, St. Louis, MO, USA). Thereafter, medium was changed daily to either IM medium or induction medium up until day 14 of differentiation.

Human embryonic kidney (HEK) 293 cells (American Type Culture Collection, Manassas, VA, USA) were cultured on tissue culture-treated dishes in HEK medium (DMEM/F-12 + Glutamax^TM^ supplemented with 10% foetal bovine serum (FBS) (Thermo Fisher Scientific, Waltham, MA, USA)). Cells were passaged every two to four days using TRYPLE (1x; Gibco, Thermo Fisher Scientific, Waltham, MA, USA).

### Generating H9-*FOXL2*-DDdCas9VP192 hESCs

The H9-*FOXL2*-DDdCas9VP192 hESCs were generated following the method described by Sepponen et al., 2022 [14]. CRISPRa guide RNAs (gRNAs) were designed using the online gRNA design tool Benchling (San Francisco, CA, USA) or selected from a pre-existing human CRISPRa pooled library [15]. A total of ten sequences targeting a region − 50 to -500 bp from the transcriptional start site in the *FOXL2* promoter were selected. From those, five gRNAs (gRNAs 2, 5, 6, 7 and 8) with the best off-target score were chosen to assemble into *FOXL2* gRNA-PCR cassettes. The gRNA cassettes consisted of a 19 bp sequence that matched the U6 promotor sequence, the 20 bp gRNA (with or without an extra guanine) and a 19 bp tailed terminator sequence. Thus, the gRNA cassette of roughly 60 bp was of the following format: 5’-TGGAAAGGACGAAACACCgNNNNNNNNNNNNNNNNNNNNgttttagagctagaaatag-3’.

The cassettes were assembled by PCR amplification as described by Balboa et al. [16]. Next, individual gRNAs and combinations of two gRNAs were tested in HEK293 cells. For this, HEK293 cells were seeded 24h prior to transfection onto a 0.1% gelatine (Sigma-Aldrich, Burlington, MA, USA)-coated 24-well plate at a density of 9 × 10^5^ cells/well in HEK medium. Cells were transfected with 500ng of the pCXLE-dCas9-VP192-T2A-EGFP-shP53 plasmid (RRID: Addgene_69535, Balboa et al., 2015 [16]) and 200ng of gRNA transcriptional unit using the FuGENE HD transfection reagent (Promega, Madison, WI, USA) in Opti-MEM medium (Gibco, Thermo Fisher Scientific, Waltham, MA, USA). Positive control cells were transfected with a pool of all gRNA transcriptional units and negative control cells were transfected with the GG-EBNA-TdT-g1-PGK-Puro plasmid (RRID: Addgene_102903, Weltner et al., 2018 [17]). Testing was performed in triplicate wells and after 72h cells were collected to determine *FOXL2* gene expression levels.

The optimal gRNA combination (gRNA 2 and gRNA 8) was concatenated using Golden Gate Assembly [18] into a GG-dest vector (RRID: Addgene_69538, Balboa et al., 2015 [16]). The assembly of the guide reactions, subsequent transformation of the reaction products into DH5α chemically competent bacteria (New England Biolabs, Ipswich, MA, USA), and screening of positive colonies were performed as described by Balboa et al., 2015 [16]. Successful concatenation of the gRNA-PCR products into the vector was confirmed by Sanger sequencing (Eurofins Genomics, Köln, Germany). Next, the correct concatenated guides were subcloned into a PiggyBac (PB) plasmid using the Epstein-Barr virus nuclear plasmid GG-EBNA-TdT-guide1-PGK-Puro (Addgene_102903; Weltner et al., 2018 [17]). The PB backbone was obtained from the PB-GG-MIR302-7 g-PGK-Puro plasmid.

Next, the PB plasmid containing the two gRNAs targeting the *FOXL2* promotor was electroporated into H9 activator cells containing SB-tight-DDdCas9VP192-GFP-Zeo-WPRE and SB-CAG-rtTA-IN-IRES-Neo with the Neon™ Transfection System (Invitrogen, Thermo Fisher Scientific, Waltham, MA, USA) following the protocol from the Neon transfection system 100 µl kit (Invitrogen, Thermo Fisher Scientific, Waltham, MA, USA). Cells were incubated with 10 µM ROCKi for four hours before transfection. Next, cells were dissociated into a single-cell suspension using TRYPLE (1x). 1 × 10^6^ cells were counted and resuspended in 100 µl R-buffer. The cell suspension was then mixed with the plasmid mix containing 1000 ng PB-plasmid containing the guides and 500 ng of the PB-transposase pCMV-Hahg-PBase. Subsequently, the transfection was performed with electroporation settings set at 1100 V, 20ms, 2 pulses. Post-electroporation, cells were plated onto a Geltrex™ LDEV-Free Reduced Growth Factor Basement Membrane Matrix-coated 10 cm dish in E8 medium containing 10 µM ROCKi. Cells were allowed to recover for three days with medium changes every other day. After this, cells were selected using 0.5 µg/ml Puromycin (Gibco, Thermo Fisher Scientific, Waltham, MA, USA) for 24h. All plasmids and the H9 activator cells were kindly provided by the Biomedicum Stem Cell Centre.

gRNA sequences and Golden Gate concatenation sequences are listed in Tables 1 and 2, respectively.

**Table 1:** gRNA sequences

Guide	Sequence 5’ ◊ 3’
gRNA 2	GTGGAAAGGACGAAACACCGGAGATGAACTCGCCCGTGCGGTTTTAGAGCTAGAAATAG
gRNA 8	GTGGAAAGGACGAAACACCGGGGCGCGTGAGCCTGGCTGTGTTTTAGAGCTAGAAATAG

**Table 2:** Golden gate concatenation sequences

Primer	Sequence 5’ ◊ 3’	Compatibility
1 aggc Fw	ACTGAATTCGGATCCTCGAGCGTCTCACCCTGTAAAACGACGGCCAGT	GG-dest
1 aggc Rv	CATGCGGCCGCGTCGACAGATCTCGTCTCACATGAGGAAACAGCTATGACCATG	2 aggc Fw
2 aggc Fw	ACTGAATTCGGATCCTCGAGCGTCTCACATGGTAAAACGACGGCCAGT	1 aggc Rv
5 aggc Rv	CATGCGGCCGCGTCGACAGATCTCGTCTCACGTTAGGAAACAGCTATGACCATG	GG-dest

### Generating H9-*FOXL2*-DDdCas9VP192 clonal lines

H9-*FOXL2*-DDdCas9VP192 hESCs were treated with 10 µM ROCKi in E8 medium for four hours prior to dissociation. The cells were then dissociated into a single-cell suspension using TRYPLE (1x) dissociation agent and resuspended in a buffer composed of 10% FBS in PBS and centrifuged at 200 rcf for three minutes. The resulting pellet was resuspended in FACS buffer consisting of Hank’s Balanced Salt Solution (Gibco, Thermo Fisher Scientific, Waltham, MA, USA), 1 mM Ultrapure™ EDTA, 25 mM HEPES (Lonza, Basel, Switzerland), 10% FBS and 10 µM ROCKi. The cell suspension was passed through a 40 µM cell strainer (Falcon, Thermo Fisher Scientific, Waltham, MA, USA) and counted with the Countess II Automated Cell Counter (Thermo Fisher Scientific, Waltham, MA, USA). The cells were kept on ice until single-cell sorting. Cell sorting was performed using the SH800Z Cell Sorter (SONY, Minato City, Tokyo, Japan) into a 96-well plate containing room temperature (RT) E8 medium supplemented with 5 µM ROCKi, Penicillin-Streptomycin (1x; Gibco, Thermo Fisher Scientific, Waltham, MA, USA) and CloneR™ (1:10; STEMCELL Technologies, Vancouver, Canada). After sorting, the plates were centrifuged at 70 rcf for three minutes and then carefully placed in a 37 °C incubator with 5% CO_2_ for 4h. After 48h, a partial media change was performed using E8 medium supplemented with 5 µM ROCKi and CloneR (1:10). Thereafter, a complete media change was performed every other day with E8 medium supplemented with 10 µM ROCKi until the colonies could be picked and expanded.

### Real time quantitative polymerase chain reaction (RT-qPCR)

RNA isolation, reverse transcription and RT-qPCR procedures were conducted as previously described in Danti et al. [19]. Primer sequences used to perform the RT-qPCRs are listed in Table 3.

**Table 3:** RT-qPCR primer sequences

Gene	Forward 5’ ◊ 3’	Reverse 3’ ◊ 5’
*DHH*	ACCTCGTGCCCAACTACAAC	CTCCTTACAACGCTCGGTCA
*FOXL2*	TTTGTCCCCTCAGTTTATGTCC	TGA ATTTGGGCAGGAGACG
*GATA4*	CAGGCGTTGCACAGATAGTG	CCCGACACCCCAATCTC
*INHBA*	GGACATCGGCTGGAATGACT	GGCACTCACCCTCGCAGTAG
*LHX9*	GCGAACCTCTTTCAAGCATC	TCCTTCTGAATTTGGCTCGT
*NR0B1*	TGCTCTTTAACCCGGACGTG	GCGTCATCCTGGTGTGTTCA
*OSR1*	GCTGTCCACAAGACGCTACA	CCAGAGTCAGGCTTCTGGTC
*PPIG*	TCTTGTCAATGGCCAACAGAG	GCCCATCTAAATGAGGAGTTG
*RSPO1*	GCAACCCCGACATGAACAAG	CAAGCCCTCCTTACACTTGG
*SOX9*	GTAATCCGGGTGGTCCTTCT	GTACCCGCACTTGCACAAC
*TAC1*	GCCTCAGCAGTTCTTTGGATTA	GAGATCTGGCCATGTCCATAAAG
*TSPAN8*	TGGACTGGCAGTTATTGAGATAC	GGTTTGACTGACGATAGGTTGA
*UPK3B*	ATCACTCTCCACCAAGGGA	CAGAGAAGAGAGGATGGAGGTA
*WNT4*	GATGTGCGGGAGAGAAGCAA	ATTCCACCCGCATGTGTGT

Briefly, RNA was isolated from cell lysates according to the Nucleospin RNA kit (Macherey-Nagel, Düren, Nordrhein-Westfalen, Germany) protocol, excluding the genomic DNA removal steps. The genomic DNA was removed separately using RQ1 RNAse-free DNAse (Promega, Madison, WI, USA), followed by RNA purification using the Nucleospin RNA Clean-Up kit (Macherey-Nagel, Düren, Nordrhein-Westfalen, Germany). Next, RNA was reverse transcribed using moloney murine leukemia virus (M-MLV) Reverse Transcriptase (Promega, Madison, WI, USA), random hexamer primer, oligo(dt)18 primer, RiboLock RNAse Inhibitor, and a mixture of four deoxynucleotide triphosphates (all from Thermo Fisher Scientific, Waltham, MA, USA). For the RT-qPCR reaction, cDNA was combined with HOT FIREPol EvaGreen qPCR Mix Plus (Solis Biodyne, Tartu, Estonia) and 2 µM reverse primers (Metabion, Planegg/Steinkirchen, Germany). Relative messenger RNA (mRNA) expression levels were analysed using the LightCycler96 system (Roche Diagnostics, Mannheim, Germany). Next, gene expression was quantified using the ΔΔCt method [20] and normalised using peptidylprolyl isomerase G (PPIG) as the endogenous control. Lastly, expression levels were presented relative to those in undifferentiated cells.

### Immunofluorescence staining

At day 14 of differentiation, cells differentiated on 4-well µ-slides were washed once with RT PBS + Mg^2+^+Ca^2+^ and thereafter fixed with 4% paraformaldehyde for 15 minutes at RT. Post-fixation, the cells were washed three times with RT PBS (Medicago, Uppsala, Sweden). Next, fixed cells were permeabilised with 0.5% Triton^®^ X-100 (Fisher Scientific, Thermo Fisher Scientific, Waltham, MA, USA) in PBS and washed three times with 0.1% Tween^®^ (Fisher Scientific, Thermo Fisher Scientific, Waltham, MA, USA) in PBS and blocked using the UltraVision Protein Block (Fisher Scientific, Thermo Fisher Scientific, Waltham, MA, USA) at RT for 10 minutes. Subsequently, the fixed cells were incubated overnight at 4°C with primary antibodies in 0.1% PBS-Tween: goat polyclonal anti-FOXL2 (Novus Biologicals, Cat# NB100-1277, RRID: AB_2106187, 1:250), mouse monoclonal anti-GATA4 (Santa Cruz Biotechnology, Cat# sc-25310, RRID: AB_627667, 1:200), mouse monoclonal anti-DHH (Santa Cruz Biotechnology, Cat# sc-271168, RRID: AB_10608075, 1:200). After primary antibody incubation, the cells were washed three times with 0.1% PBS-Tween and incubated with the following secondary antibodies at a dilution of 1:1000 in 0.1% PBS-Tween: Alexa Fluor^®^ 488 donkey anti-goat IgG (Thermo Fisher Scientific, Cat# A-11055, RRID: AB_2534102) or Alexa Fluor^®^ 594 donkey anti-mouse (Thermo Fisher Scientific, Cat# A-21203, RRID: AB_2535789). The incubation was carried out at RT for 45 minutes in the dark. The cells were washed twice with 0.1% PBS-Tween. Nuclei were subsequently stained with 4’,6-diamidino-2-phenylindole (DAPI) dilactate (Invitrogen, Thermo Fisher Scientific, Cat# D3571, RRID: AB_2307445) at a 1:1000 ratio in 0.1% PBS-Tween. Incubation with DAPI was carried out for 10 min in the dark and afterwards cells were washed twice with 0.1% PBS-Tween.

Confocal images were captured using a TCS SP8 confocal microscope with a white laser (Leica Microsystems, Mannheim, Germany) at an 812 × 812 format with an HC PL APO CS2 40x/1.30 oil objective. Images were processed using Fiji version 2.3.0 (http://fiji.sc).

### Bulk RNA sequencing library preparation, sequencing, and data analysis

Cells cultured in triplicate in 12-well culture plates were lysed with RA1 lysis buffer. Total RNA (tRNA) was isolated from the samples using the Nucleospin RNA kit with an additional DNAse I treatment to remove genomic DNA. The RNA was subsequently purified using the Nucleospin RNA clean-up kit.

Determination of RNA quality, library preparation and sequencing were conducted by Novogene Europe in Cambridge, England. RNA quality control was performed using the Agilent 2100 bioanalyzer (Agilent, Santa Clara, CA, USA) to assess RNA integrity and purity. Following quality control, mRNA was isolated from tRNA employing poly-T oligo-attached magnetic beads (ABclonal, Düsseldorf, Germany) for mRNA library preparation. The mRNA was then fragmented and thereafter cDNA was synthesised through reverse transcription using random hexamer primers for the first strand and dUTP/dTTP for the second cDNA strand. The non-directional library was finalised after end-repair A-tailing, adapter ligation, size selection, amplification, and purification. The mRNA library was subsequently quantified using Qubit (Invitrogen, Thermo Fisher Scientific, Waltham, MA, USA) and RT-PCR. Bioanalyzer was used to check the size distribution detection. The quantified libraries were sequenced on the Illumina sequencing platform NovaSeq X Plus series with 9G raw data per sample. The read length for the paired-end run was 150 bp.

Data pre-processing was performed by Novogene. Raw reads in fastq format were processed using the fastp software (version 0.23.2) to obtain clean reads by removing reads containing adapter, poly-N and low-quality reads from the raw data. Simultaneously, the Q20, Q30 and GC content of the clean data were calculated. All the further downstream analyses were performed on high-quality clean data.

Next, the reads were mapped to the reference genome, directly obtained from the genome website, using Hisat2 (v2.0.5). Gene expression levels were quantified using featureCounts (v1.5.0-p3) and expressed as Fragments Per Kilobase of transcript sequence per Millions base pairs sequenced (FPKM). Differential expression (DE) analysis of the two conditions or groups (three technical replicates per condition/group) was performed using the DESeq2Rpackage (1.20.0). The resulting *P*-values were adjusted for false discovery rate using the Benjamini and Hochberg’s approach. Genes with an adjusted *P*-value < 0.05 found by DESeq2 were assigned differentially expressed.

The gene ontology (GO) enrichment analysis of differentially expressed genes (DEGs) was performed using the clusterProfiler R package, correcting for gene length bias. GO terms with *P*-value < 0.05 were considered significantly enriched. The same package was used for Kyoto encyclopaedia of genes and genomes (KEGG) pathway enrichment analysis of DEGs. Again, terms of which the *P*-value < 0.05 were considered significantly enriched.

### Statistics

Statical analysis for RT-qPCR was performed using the software Graphpad Prism 9 version 9.2.0 (La Jolla, CA, USA). Two-way ANOVA was performed to determine the statistical significance between two or more groups/conditions. The Sidak’s post-hoc test was used as the correction method for multiple comparisons. Statistical significance was attributed if the *P*-values were less than 0.05. Data are shown as mean ± SEM. The statistical analysis employed for the bulk RNA-seq data is explained above in the section ‘bulk RNA sequencing library preparation, sequencing, and data analysis’.

## Results

### *FOXL2* represses gonadal markers in early somatic cell gonadal differentiation

To investigate the function of *FOXL2* in early somatic cell ovarian development, a dual inducible *FOXL2* activation line - H9-*FOXL2*-DDdCas9VP192 - was established using CRISPR/Cas9-mediated genome activation. The cells were subjected to our previously established gonadal differentiation protocol [13]. To mimic the in vivo situation as closely as possible, we first optimised the day of *FOXL2* induction (Suppl. Figure 1A). As our differentiation protocol steers the cells from human pluripotent stem cells (hPSCs) to intermediate mesoderm (IM) during the first four days of differentiation, we selected an early and a late time point: day 4 and day 8, respectively and followed the differentiation up to day 14 (Suppl. Figure 1A).

**Fig. 1:**
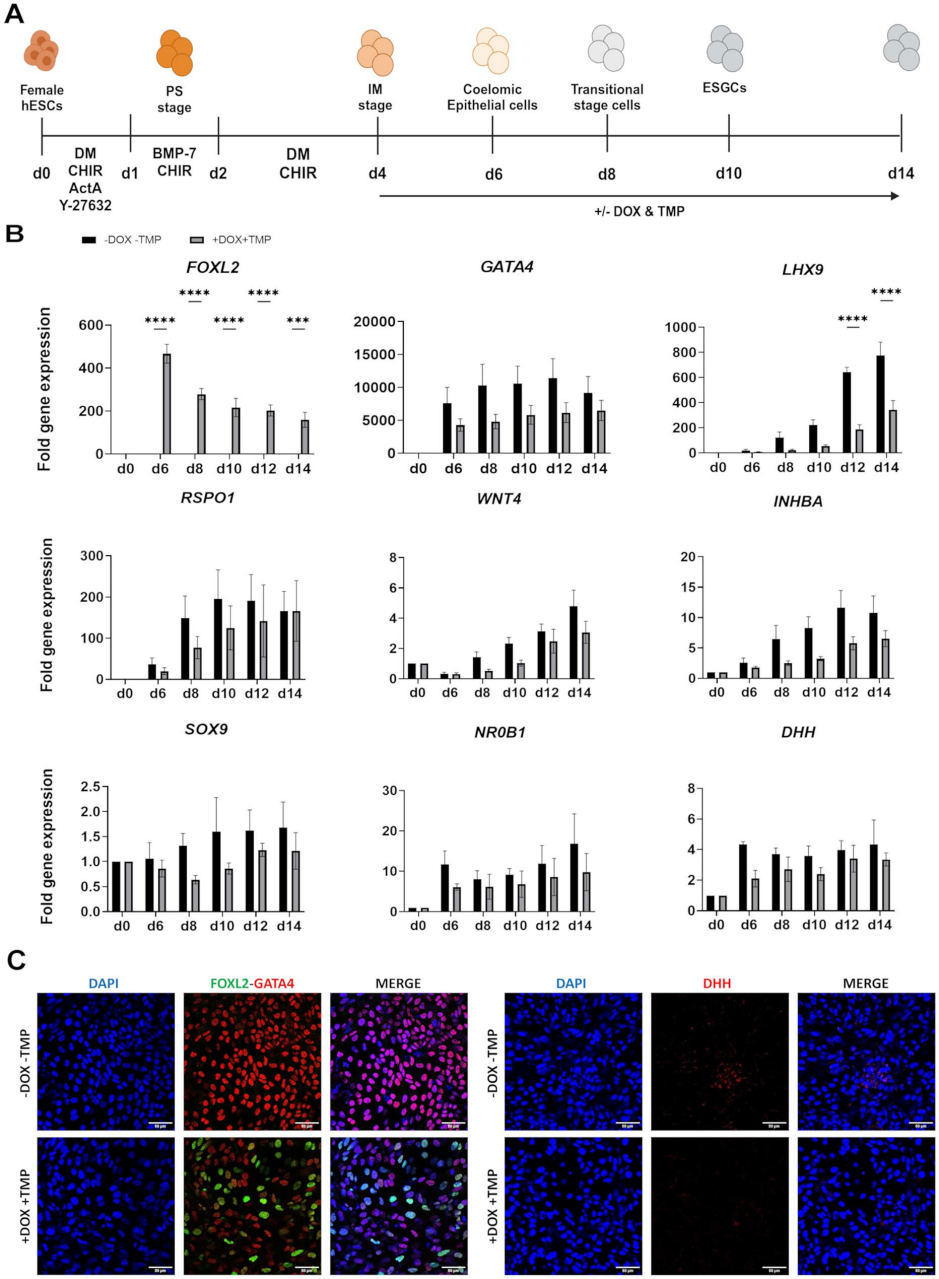
*FOXL2* is a repressive factor when induced at day 4 of gonadal differentiation. (**A**) A schematic representation of the 14-day gonadal differentiation protocol with the added growth factors, inhibitors and small molecules used to steer female hESCs towards ESGCs and the respective developmental stages throughout differentiation. *FOXL2* is induced at day 4 through the addition of DOX and TMP for 10 days as shown by the arrow. Created in https://BioRender.com. (**B**) RT-qPCR analysis of *FOXL2* induction at day 4 of gonadal differentiation. *FOXL2* was induced through the addition of DOX and TMP and downregulated gonadal markers *GATA4*, *LHX9*, *RSPO1*, *WNT4*, *INHBA*, *SOX9*, *NR0B1* and *DHH*. The fold change is presented in comparison to d0 (undifferentiated cells) gene expression levels. Data are reported as mean ± SEM, *n* = 4 biological replicates. Two-way ANOVA; 0.1234 (ns), 0.0332 (*), 0.0021 (**), 0.0002 (***), 0.0001 (****). (**C**) Confocal images of IF staining show expression of FOXL2, GATA4 and DHH in the non-induced (-DOX -TMP) and in the induced (+ DOX + TMP) conditions at day 14 of gonadal differentiation. Images were taken with an 40X objective. Scale bars 50 μm. ActA, activin A; BMP, bone morphogenetic protein; CHIR, CHIR-99021; DM, dorsomorphin; ESGCs, early supporting gonadal cells; hESCs, human embryonic stem cells; IM, intermediate mesoderm; PS, primitive streak; d, day of differentiation; DOX, doxycycline hyclate; TMP, trimethoprim

We first studied the effect of *FOXL2* induction at day 4 during the gonadal differentiation process (Fig. 1A). RT-qPCR analysis showed that upon induction, the expression levels of *FOXL2* were upregulated by an average of 200-fold. Subsequent RT-qPCR analyses revealed that *FOXL2* induction initiated at day 4 resulted in a significant downregulation of the gonadal marker LIM-homeobox 9 (*LHX9*) and in a slight reduction in the gene expression levels of several early gonadal markers. These markers included the coelomic epithelial markers GATA-binding protein 4 (*GATA4*), the female gonadal markers *RSPO1* and *WNT4*, the male gonadal markers SRY-box transcription factor 9 (*SOX9*), desert hedgehog (*DHH*) and nuclear receptor subfamily 0 group B member 1 (*NR0B1*), as well as the gonadal marker inhibin subunit beta a (*INHBA*) (Fig. 1B). Immunofluorescence (IF) co-staining of FOXL2 and GATA4 on day 14 of gonadal differentiation showed the absence of FOXL2 in the control cultures, whereas most of the cells were GATA4 positive. In contrast, in the induced state, FOXL2 protein was clearly detectable and GATA4 expression at the protein level was decreased. Notably, even though not all cells expressed FOXL2, possibly due to gene silencing, the difference in GATA4 expression between the control and induced states was more pronounced at the protein level than at the RT-qPCR level. Additionally, IF staining of DHH showed a similar decreased expression pattern at the protein level as seen with GATA4 (Fig. 1C).

RT-qPCR analyses indicated that the addition of doxycycline (DOX) and trimethoprim (TMP) at day 8 of gonadal differentiation elevated *FOXL2* gene expression levels approximately by 300-fold on average until day 14. However, *FOXL2* induction at this later time point did not appear to affect the expression levels of coelomic epithelial markers *GATA4* and *LHX9* at any timepoint. Additionally, the gene expression levels of female gonadal markers *RSPO1*, *WNT4* and *INHBA* were only slightly downregulated following *FOXL2* induction at day 8 (Suppl. Figure 1B).

In summary, the earlier *FOXL2* is induced during differentiation, the more significant its impact on the expression levels of gonadal markers. Therefore, we chose day 4 as the induction time point for the remainder of our study. Moreover, we can conclude that in the earlier stages of gonadal development, *FOXL2* induction seems to primarily exert a repressive effect on coelomic epithelial, female, and male gonadal markers.

### Transcriptional changes are induced by *FOXL2* during early gonadal differentiation

To further study the role of *FOXL2* during early somatic cell gonadal differentiation in more detail, we opted to perform bulk RNA sequencing (RNA-seq) analyses on the non-induced (CTRL, -DOX -TMP) and induced (IND, +DOX + TMP) cells. Samples were collected on day 4 and then every two days (day 6, 8, 10, 12, 14) in three technical replicates per timepoint. According to the principal component analysis (PCA), 33.43% of the variance between the samples could be attributed to the process of gonadal differentiation (principal component 1, PC1), while *FOXL2* induction (principal component 2, PC2) accounted for 16.7% of the observed variance. Not only was there a clear divergence between the conditions, but PC1 also indicated the divergence between the different days of gonadal differentiation. Day 4, 6, 8 and 10 samples were distinct from each other, reflecting differences in developmental state. However, the day 12 and 14 samples clustered more tightly together, indicating greater similarity between these developmental stages (Suppl. Figure 2 ).

**Fig. 2:**
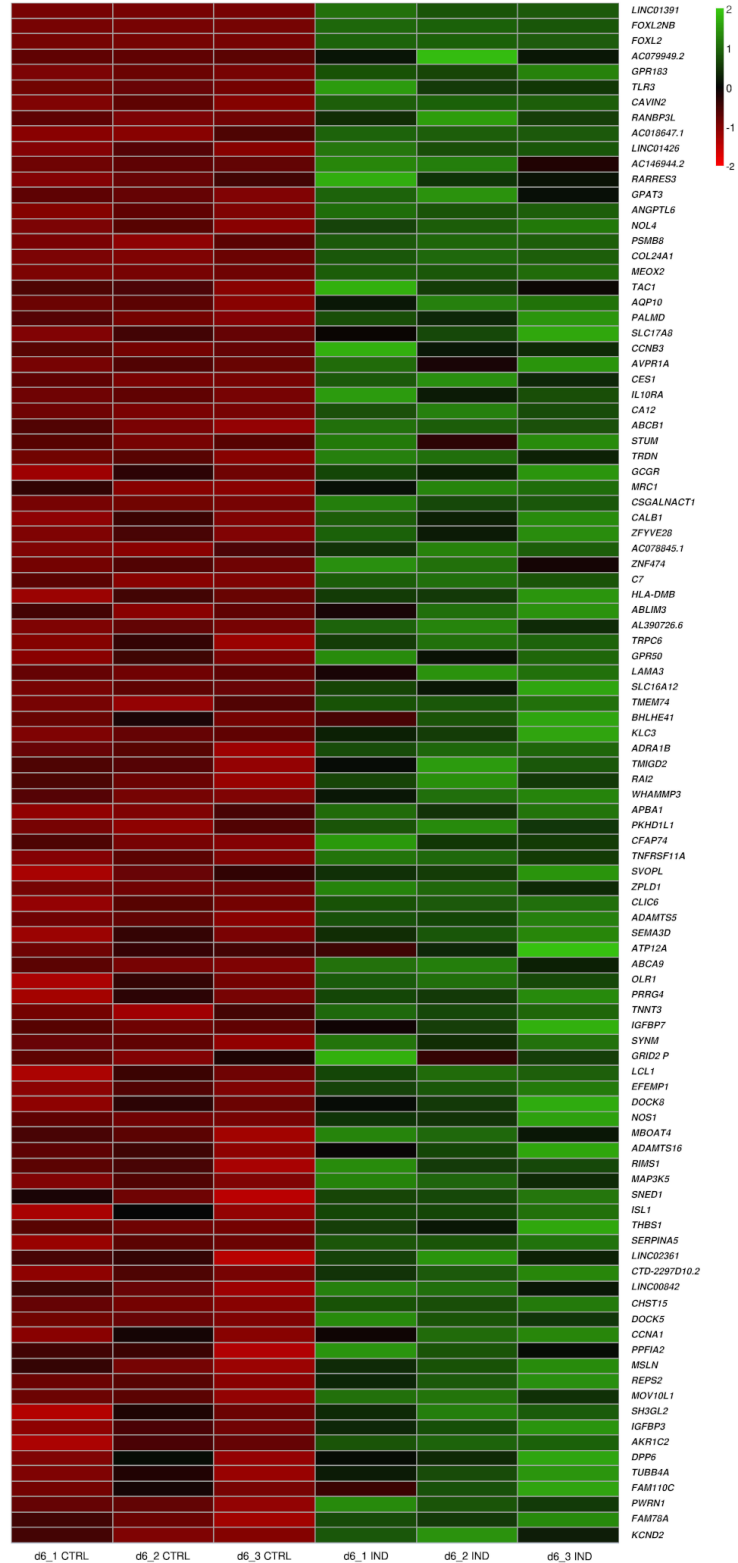
Expression pattern of the top 100 upregulated differentially expressed genes (DEGs) at day 6 of differentiation upon *FOXL2* induction. The heat map is showing the comparison between the control (CTRL, -DOX–TMP) and induced (IND, +DOX + TMP) conditions at 6 day of gonadal differentiation. The top 100 DEGs at day 6 of differentiation are displayed and each rectangle represents the expression of a specific gene within a technical replicate. The intensity of gene expression is indicated by a colour scale based on a log2 scale (red = lowest expression, green = highest expression). d, day

Next, we performed pairwise differential expression (DE) comparisons to assess the immediate effect of *FOXL2* on the cells, focusing on the day 6 non-induced (CTRL, -DOX -TMP) versus day 6 induced (IND, +DOX + TMP) conditions. To do this, we plotted a heatmap representing the expression profile of the top 100 upregulated DEGs with the highest Log2FoldChange at day 6 of gonadal differentiation. *LINC01391*, along with FOXL2 neighbour (*FOXL2NB*) and *FOXL2*, were the top three upregulated genes at this time point. Other notable genes among the top 100 upregulated DEGs include toll-like receptor 3 (TLR3) and tachykinin precursor 1 (*TAC1*) (Fig. 2).

Next, we looked at the Kyoto encyclopaedia of genes and genomes (KEGG) and gene ontology (GO) pathways associated with the upregulated DEGs at day 6 of gonadal differentiation. The upregulated DEGs were associated with KEGG pathways such as phagosome, neuroactive ligand-receptor interaction, complement and coagulation cascades and cell adhesion molecules (Suppl. Figure 3 A). As for the GO pathways, DEGs were mostly associated with hormone regulation and interferon response regarding biological processes (BP), epithelial junction formation and plasma membranes in terms of cellular component (CC) and lastly enzyme activity and hormone binding as molecular functions (MF). We further examined the top up-and downregulated DEGs at each time point to determine the identity of the cells after *FOXL2* induction. The upregulated DEGs were mostly associated with neuroactive ligand-receptor interactions and cell adhesion molecules KEGG pathways as well as similar GO pathways as seen at day 6 (Suppl. Figure 3B).

Overall, the upregulated DEGs are primarily linked with KEGG pathways involving cell adhesion molecules and GO pathways relating to extracellular matrix and junction formation at different time points during gonadal differentiation.

### *FOXL2* supports the transition from coelomic epithelium to early supporting gonadal cells

**Fig. 3:**
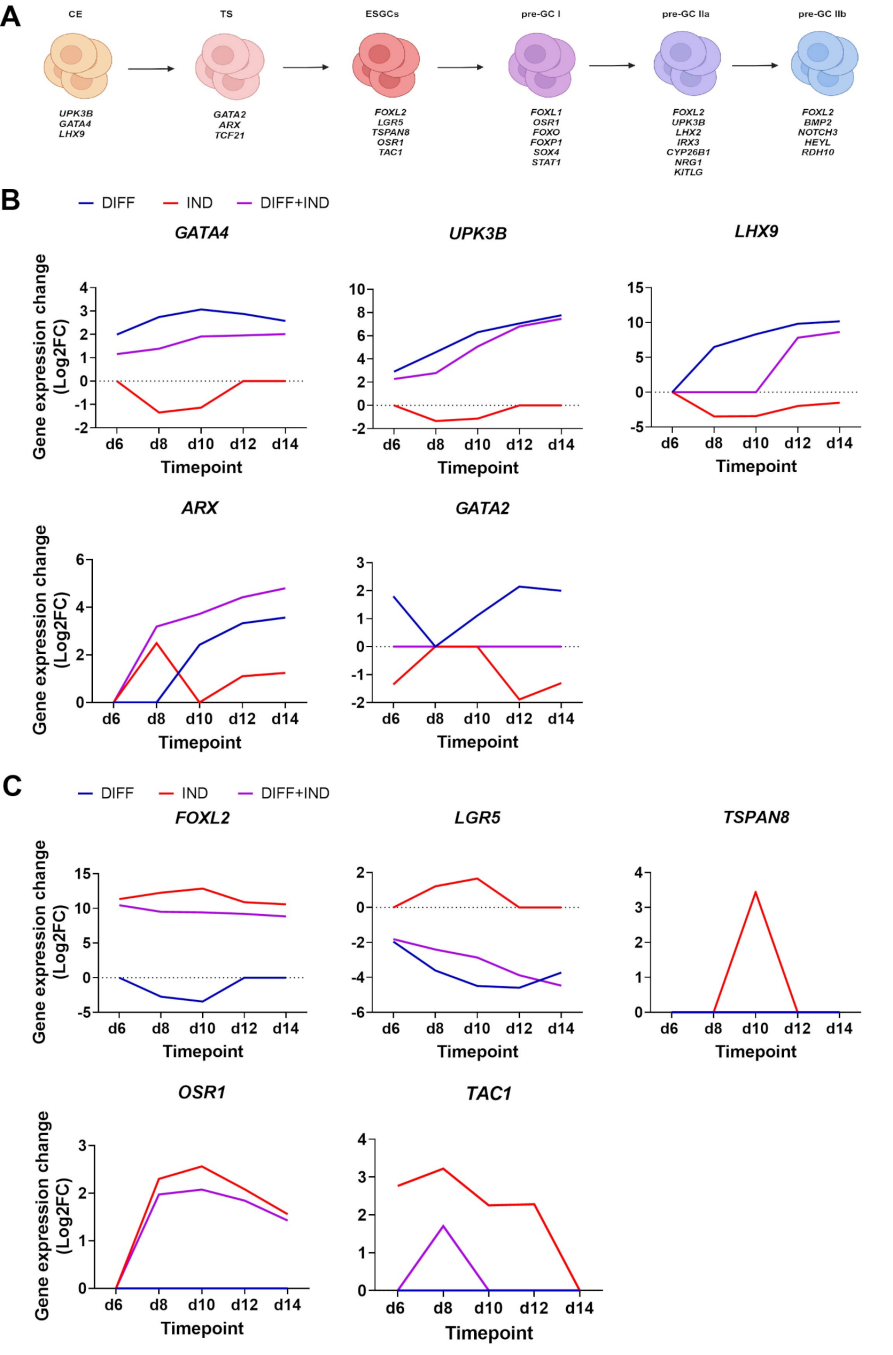
*FOXL2* drives the cells out of a coelomic epithelial fate into a transitional stage and finally to an early supporting gonadal cell fate. (**A**) Schematic representation of early gonadal development as described by Garcia-Alonso et al. as well as the matching developmental stages and their associated marker genes according to both Garcia-Alonso et al. and Wamaitha et al. Created in https://BioRender.com. (**B** and **C**) Line graphs of marker genes associated with the coelomic epithelial, transitional stage and ESGCs. Gene expression change is shown for three conditions: the effect of gonadal differentiation (DIFF, blue), the effect of *FOXL2* induction (IND, red) and the combined effect (DIFF + IND, purple). Graphs show a downregulation of *GATA4*, *LHX9*, *UPK3B* from day 6 of gonadal differentiation, an upregulation of *ARX* and *GATA2* at day 8 and downregulation again at day 10. ESGC markers *LGR5*, *TSPAN8*, *OSR1* and *TAC1* are upregulated upon *FOXL2* induction at day 10. *N* = 1 biological replicate with 3 technical replicates. CE, coelomic epithelium; ESGCs, early supporting gonadal cells; GC, granulosa cell; TS, transitional stage

Next, we turned to the literature and compared our data to data from two relevant publications focusing on in vivo early gonadal development: the study of Garcia-Alonso et al., published in Nature in 2022 [21]; and the study by Wamaitha et al., published in Developmental Cell in 2023 [22]. Both studies conducted single-cell RNA-seq analysis of early human gonadal tissue (approximately 6–21 post-conception week (PCW)). The Garcia-Alonso study mapped the trajectory of somatic cell gonadal differentiation all the way from coelomic epithelium to pre-granulosa cells or Sertoli cells, identifying specific maker genes for each differentiation stage. Similarly, the Wamaitha et al. study detailed subsequent stages of early somatic cell gonadal differentiation, with marker genes that further corroborated the findings of Garcia-Alonso et al. (Fig. 3A).

To compare our RNA-seq data with those from the two previous studies, we performed pairwise DE comparisons. The data presented in the graphs are based on the mean of three technical replicates of one biological replicate. First, we studied the effect of gonadal differentiation (DIFF) only on the cells by comparing the day 4 samples with the non-induced (CTRL, -DOX -TMP) samples at each time point to assess the progression and identity of the differentiating cells without *FOXL2* activation. We observed that without *FOXL2*, the coelomic epithelial marker genes *GATA4* and uroplakin 3b (*UPK3B*) were upregulated at day 6, with *LHX9* appearing just after day 6, and aristaless related homeobox (*ARX*) and GATA binding protein 2 (GATA2) at day 10. From day 10 onwards, all coelomic epithelial markers stayed upregulated. Secondly, we analysed the effect of *FOXL2* induction (IND) only on the cells by comparing the non-induced (CTRL, -DOX -TMP) and induced (IND, +DOX + TMP) samples at each specific time point during differentiation. The aim was to examine the sole effect of *FOXL2* activation to determine its role in early somatic cell gonadal differentiation and determine cell identity at each time point during differentiation. At day 6, only *FOXL2* was differentially expressed and upregulated due to the activation. However, by day 8, three important coelomic epithelial markers-*GATA4*, *UPK3B* and *LHX9*-were downregulated, whilst *ARX*, *GATA2* and *FOXL2* were upregulated. At day 10, the coelomic epithelial genes remained downregulated and *FOXL2* upregulated. Additionally, the early supporting gonadal cell (ESGC) markers leucine rich repeat containing G protein-coupled receptor 5 (*LGR5*), tetraspanin 8 (*TSPAN8*), odd-skipped related transcription factor 1 (*OSR1*) and *TAC1* were upregulated for the first time alongside *FOXL2*. ARX was no longer differentially expressed at day 10. From day 10 onwards, *OSR1* remained upregulated alongside *FOXL2*. *LHX9* was downregulated at day 12 whilst ARX was upregulated again. At day 14 *LHX9* was still downregulated and *ARX* upregulated. Lastly, we examined the combined effect of gonadal differentiation and *FOXL2* induction (DIFF + IND) on the cells by comparing the day 4 non-induced (CTRL, -DOX -TMP) samples with the induced (IND, +DOX + TMP) samples at each specific time point. The DEGs in this comparison shared similarities with both the differentiation only (DIFF) and *FOXL2* induction only (IND) comparisons. However, they predominantly resembled the sole effect of gonadal differentiation (DIFF). The most notable difference from the differentiation only (DIFF) comparison was the upregulation of both *FOXL2* and *OSR1*. The main difference from the induction only (IND) comparison was the consistent downregulation of *LGR5* across all time points (Fig. 3B and C).

Next, we validated our bulk RNA-seq data through RT-qPCR analysis of key marker genes from each developmental stage. The validation showed that *UPK3B* was downregulated and *TSPAN8*, *OSR1*, and *TAC1* gene expression levels were upregulated upon *FOXL2* induction. Generally, the genes behaved similarly as in our bulk RNA-seq data, further supporting the results (Suppl. Figure 4).

Thus, *FOXL2* induction appears to guide cells from a coelomic epithelial identity towards the ESGC stage and potentially towards pre-granulosa cells, making *FOXL2* not only a marker but also a potential driver of the formation of ESGCs.

## Discussion

In this study, we demonstrate that *FOXL2* is not just a marker of ESGCs but plays a role in the transition of cells from a coelomic epithelial state to ESGCs. Utilising CRISPR/Cas9-mediated genome activation of *FOXL2*, we observed the downregulation of several established coelomic epithelial markers including *GATA4*, *LHX9* and *UPK3B* starting around day 6 of gonadal differentiation. The downregulation of these markers signified the cells’ exit from the coelomic epithelial fate by approximately day 8 of gonadal differentiation, at which point transition markers such as *ARX* and *GATA2* became upregulated. These markers became subsequently decreased after day 8, followed by the upregulation of ESGC markers, including *LGR5*, *TSPAN8*, *TAC1* and *OSR1*, by day 10.

Our bulk RNA-seq data supported our initial findings that *FOXL2* is involved in repressing several factors during early gonadal somatic cell differentiation. According to the KEGG and GO pathway analysis, the upregulated DEGs at day 6 were primarily associated with cell adhesion molecules, extracellular matrix, and junction formation. These pathways remained relevant across different time points and developmental stages throughout gonadal differentiation, indicating changes at the cell identity level of the differentiating cells. Using data from Garcia-Alonso et al. [21] we were able to assign labels to these developmental stages. As coelomic epithelial markers were still differentially expressed and upregulated at day 6 of gonadal differentiation, we hypothesized that the cells at this stage were coelomic epithelial-like. However, shortly thereafter, *FOXL2* began downregulating the coelomic epithelial markers *GATA4*, *LHX9*, and *UPK3B*. By day 8 of gonadal differentiation markers for the genital ridge stage, such as *ARX* and *GATA2*, were upregulated. This signifies a shift in cell identity from the coelomic epithelial to the transitional stage, as observed in the data from Garcia-Alonso et al. and Wamaitha et al. [21, 22]. Subsequently, we observed downregulation of *ARX* and *GATA2* gene expression, while markers for ESGCs, including *LGR5*, *TSPAN8*, *OSR1* and *TAC1*, were upregulated, indicating another shift in cell identity from the transitional stage to ESGC-like stage by day 10 of gonadal differentiation. After day 10, *OSR1* remained upregulated for the remainder of the gonadal differentiation process. The *OSR1* cell population represents precursors of the pre-GCI population during the first wave of granulosa cell formation [21]. Based on this information, we conclude that cells from day 4 until day 10 of gonadal differentiation correspond to the in vivo gonadal developmental period spanning 6–8 PCW. Following day 10, as *OSR1* remained upregulated, we hypothesise that our gonadal differentiation protocol combined with *FOXL2* induction could potentially steer the cells to a pre-GCI population. Additionally, Taelman et al. conducted a study using single-cell transcriptomics to characterise the foetal female and male gonads. In this study, the researchers identified several clusters of cell populations and assigned marker genes to each cell population. Clusters 5 and 12 were annotated as being coelomic/ovarian surface epithelium, while clusters 4 and 9 were annotated as pre-granulosa cells. Our cells at around days 4–6 expressed DEGs corresponding to the coelomic/ovarian surface epithelium cell population by Taelman et al., whereas our day 10–14 cells had a resemblance to the pre-granulosa cell population [23].

*FOXL2* induction appeared to actively repress male gonadal differentiation by downregulating male gonadal markers *SOX9*, *NR0B1* and *DHH*. Although the reduction in the expression levels of these male gonadal markers was only moderate, their baseline levels were already quite low, which is expected given that we are working with a female hESC line. Interestingly, *FOXL2* induction also seemed to downregulate the female gonadal markers *RSPO1* and *WNT4*. This may seem counterintuitive, as the expression of the pro-ovarian factors *RSPO1* and *WNT4* would be expected to increase during granulosa cell differentiation. However, in humans, a second wave of granulosa cells (preGC-IIa/b) emerges after 8 PCW, during which *RSPO1* and *WNT4* are actively downregulated [21]. Whether *FOXL2* is a key player in that second wave of granulosa cell formation remains to be elucidated. Moreover, the study by Migale et al. showed that *FOXL2* binds and downregulates the expression levels of *RSPO1* and *WNT4* in mice [24].

Each developmental stage is represented by specific marker genes. In our study, some of the known and expected markers were not highly up- or downregulated, nor were they found among the top 100 DEGs at their respective time points. This is likely due to the presence of a heterogeneous cell population at each stage. The FOXL2/GATA4 IF staining on day 14 of differentiation showcases this heterogeneity as there are cells co-expressing FOXL2 and GATA4 whilst other cells solely express only one of the proteins. This heterogeneity could be attributed to some cells losing their *FOXL2* expression during the gonadal differentiation process. However, the cells that do not express *FOXL2* are still being subjected to our gonadal differentiation protocol, which efficiently upregulates bipotential markers such as *GATA4*. This might explain the presence of cells solely expressing GATA4 protein. Cells that retain *FOXL2* expression will solely express FOXL2 or co-express FOXL2 and GATA4. Although the reason behind why some cells solely express FOXL2 is unclear, there are several possible explanations: *FOXL2* might be repressing *GATA4* expression in these cells, or the *FOXL2*-positive cells might belong to the percentage of cells that do not upregulate *GATA4* during the differentiation. Alternatively, these FOXL2-positive cells may have differentiated into another cell type than the double positive cells. Single-cell RNA sequencing would allow us to determine which specific subpopulations are present at each time point during gonadal differentiation and their relative proportions. Another method that could be employed to characterise the different cell populations would be a flow sorting-based strategy where FOXL2/GATA4-expressing cells could be isolated, and the gene expression levels of these cell populations could be compared. It is important to note that heterogeneity is not necessarily a negative phenomenon. In vivo, multiple distinct somatic cell populations exist at each developmental stage. For instance, the study by Guo et al. showed highly heterogeneous gene expression patterns within somatic cell populations using single-cell RNA-seq analysis [25].

Among the expected granulosa cell precursor population markers, wnt family member 6 (*WNT6*), should be upregulated at the transitional stage [21]. However, in our list of DEGs, *WNT6* was found to be downregulated across all time points (data not shown). In mice, ovarian pre-granulosa cells are thought to originate from either bipotential precursor cells or surface epithelium cells. If they arise from the bipotential cells, these precursors are marked by the expression of *WNT6*, *WNT4* and *FOXL2*. If they on the other hand arise from the epithelial cells, the markers *UPK3B*, *LGR5* and keratin 19 (*KRT19*) tend to be highly expressed [26]. A similar situation may be present in humans. Our precursors most likely originate from coelomic epithelial-like cells, which would explain the downregulation of *WNT4* and *WNT6*, alongside the high expression of *UPK3B*, and *LGR5* that we observe later in our granulosa cell precursors.

*FOXL2* has known and putative direct targets, including follistatin (*FST*), cytochrome P450 family 19 subfamily A member 1 (*CYP19A1*), steroidogenic acute regulatory protein (*STAR*), cytochrome P450 family 17 subfamily A member 1 (*CYP17A1*) [27–30]. However, RT-qPCR results show no upregulation of these target genes after *FOXL2* induction (data not shown). We reason that *FOXL2* presumably has different target genes depending on the developmental stage. As we are focusing on very early gonadal development (6–8 PCW), these target genes are simply not yet expressed at the stage of pre-granulosa cell development but are expected to become active later in granulosa cell development, maturation, and the initiation of steroidogenesis. This reasoning is supported by the study by Migale et al. where the researchers looked at *FOXL2* binding partners at different timepoints during murine ovarian development. The study showed that *FOXL2* binds different target genes depending on the developmental stage and that it regulates more targets postnatally [24].

The principal strength of this paper is that it is the first study to demonstrate that *FOXL2* is not merely a marker of ESGCs but plays a potential role in their formation. Moreover, by combining growth factors, inhibitors, small molecules and CRISPR/Cas9 activation, we successfully replicated in vitro a very early in vivo stage of gonadal development (6–8 PCW) of pre-granulosa cell formation, encompassing all the key developmental stages. This model provides a valuable tool for studying the underlying pathological mechanisms of human conditions caused by *FOXL2* mutations, such as BPES and DSD. One limitation is the heterogeneity of the cells at each developmental stage despite the use of small molecules, growth factors, inhibitors, and CRISPR/Cas9 activation to guide differentiation in the desired direction. Another limitation is that the results are based solely on in vitro experiments. For future directions, it would be interesting to identify growth factors and/or small molecules that could replace the use of CRISPR/Cas9 to endogenously upregulate *FOXL2*. This could be particularly beneficial for applying this differentiation protocol in a clinical setting.

## Conclusions

In conclusion, our study indicates that during gonadal differentiation of a female hPSC line, *FOXL2* is not only a marker of ovarian development but seems to play an active role in the transition from coelomic-epithelial cells to ESGCs in early ovarian differentiation. Furthermore, we have confirmed that even during these early stages, *FOXL2* actively represses the expression of genes that are known to drive male sex differentiation. The findings in this study could aid in the elucidation of the mechanisms underlying human foetal ovarian development.

## Electronic Supplementary Material

Below is the link to the electronic supplementary material.

Supplementary Figure 1: *FOXL2* induction optimisation showing that day 8 is a sub-optimal induction day. (**A**) A schematic representation of the 14-day gonadal differentiation protocol including the different small molecules, growth factors, inhibitors used to steer female hESCs towards the IM stage and with the matching developmental stages. Arrows showing the start and end of the *FOXL2* induction through addition of the antibiotics DOX and TMP. Created in https://BioRender.com. (**B**) RT-qPCR analysis of *FOXL2* induction at day 8 of gonadal differentiation. *FOXL2* was upregulated through the addition of DOX and TMP and minimally downregulated the gonadal markers *GATA4*, *LHX9*, *RSPO1*, *WNT4* and *INHBA*. Data are reported as mean ± SEM, *n* = 3 biological replicates. The fold change is presented in comparison to d0 (undifferentiated cells) gene expression levels. Two-way ANOVA; 0.1234 (ns), 0.0332 (*), 0.0021 (**), 0.0002 (***), 0.0001 (****). ActA, activin A; BMP, bone morphogenetic protein; CHIR, CHIR-99021; DM, dorsomorphin; hESCs, human embryonic stem cells; IM, intermediate mesoderm; PS, primitive streak; d, day of differentiation; DOX, doxycycline hyclate; TMP, trimethoprim

Supplementary Figure 2: Bulk RNA-sequencing showed differences between the control (-DOX-TMP) and induced (+ DOX + TMP) conditions and between the different time points. Principal component analysis showing the divergence between samples from the different timepoints of gonadal differentiation and between control (CTRL) and induced (IND) conditions. Principal component 1 shows the effect of gonadal differentiation and principal component 2 shows the effect of *FOXL2* induction. d; day of differentiation; PC, principal component

Supplementary Figure 3: *FOXL2* induction upregulates pathways associated with cell adhesion, extracellular matrix, and junctions. (**A**) Bar charts showing Kyoto encyclopaedia of genes and genomes (KEGG) pathways associated with upregulated differentially expressed genes (DEGs) at different time points during the gonadal differentiation. Numbers above bars signify the number of genes associated with the specific pathway. (**B**) Bar charts showing gene ontology (GO) pathways associated with the upregulated DEGs at different time points during the differentiation. Numbers above the bars signify the number of genes associated with the specific pathway. Bar charts are divided into three categories: biological process (BP), cellular component (CC) and molecular function (MF)

Supplementary Figure 4: RT-qPCR validation confirms bulk RNA-seq results. RT-qPCR analysis shows the downregulation of coelomic epithelial marker *UPK3B* and the upregulation of ESGC markers *TSPAN8*, *OSR1* and *TAC1* upon *FOXL2* induction at day 4 of gonadal differentiation. The fold change is presented in comparison to d0 (undifferentiated cells) gene expression levels. Data are reported as mean ± SEM, *n* = 4 biological replicates. Two-way ANOVA; 0.1234 (ns), 0.0332 (*), 0.0021 (**), 0.0002 (***), 0.0001 (****)

## Data Availability

The datasets generated and/or analysed during the current study are available in the NCBI Gene Expression Omnibus (GEO) repository and can be accessed through the GEO series accession number GSE282662. All material unique to this study is available from the corresponding author on reasonable request.

## Abbreviations

ActA: Activin A
ARX: Aristaless related homeobox
BMP7: Bone morphogenic protein 7
BP: Biological process
BPES: Blepharophimosis, ptosis epicanthus and inversus syndrome
Cas9: CRISPR associated protein 9
CC: Cellular component
CRISPR: Clustered regularly interspaced short palindromic repeats
CTRL: Control
CYP17A1: Cytochrome P450 family 17 subfamily A member 1
CYP19A1: Cytochrome P450 family 19 subfamily A member 1
DAPI: 4’,6-diamidino-2-phenylindole
DE: Differential expression/differentially expressed
DEG: Differentially expressed genes
DHH: Desert hedgehog
DM: Dorsomorphin
DOX: Doxycycline hyclate
DSD: Differences of sex development
E8: Essential 8
EDTA: Ethylenediaminetetraacetic acid
ESGC: Early supporting gonadal cell
FBS: Foetal bovine serum
FOXL2: Forkhead box L2
FOXL2NB: FOXL2 neighbour
FST: Follistatin
GATA2: GATA binding protein 2
GATA4: GATA binding protein 4
GG: Golden gate
GO: Gene ontology
gRNA: Guide RNA
hESC: Human embryonic stem cell
HEK: Human embryonic kidney
hiPSC: Human induced pluripotent stem cell
hPSC: Human pluripotent stem cell
IF: Immunofluorescence
IM: Intermediate mesoderm
IND: Induced
INHBA: Inhibin subunit beta a
KEGG: Kyoto encyclopaedia of genes and genomes
KO: Knock-out
KRT19: Keratin 19
LHX9: LIM homeobox 9
LGR5: Leucine rich repeat containing G protein-coupled receptor 5
MF: Molecular function
mRNA: Messenger RNA
NR0B1: Nuclear receptor subfamily 0 group B member 1
OSR1: Odd-skipped related transcription factor 1
PB: Piggyback
PBS: Phosphate-buffered saline
PC: Principal component
PCA: Principal component analysis
PCW: Post conception week
PIS: Polled intersex syndrome
PPI: Protein-protein interaction
PPIG: Peptidylprolyl isomerase G
Pre-GC: Pre-granulosa cell
PS: Primitive streak
RT: Room temperature
RT-qPCR: Real time quantitative polymerase chain reaction
ROCKi: Rho-kinase inhibitor
RSPO1: R-spondin 1
SOX9: SRY-box transcription factor 9
STAR: Steroidogenic acute regulatory protein
TAC1: Tachykinin precursor 1
TMP: Trimethoprim
tRNA: Total RNA
TSPAN8: Tetraspanin 8
UPK3B: Uroplakin 3B
WNT4: Wnt family member 4
WNT6: Wnt family member 6
