# Supplementary figures and images for: FOXL2 drives the differentiation of supporting gonadal cells in early ovarian development
Source: Reprod Biol Endocrinol. 2025 Mar 18;23:44. doi: 10.1186/s12958-025-01377-0 (PMC11917015; doi:10.1186/s12958-025-01377-0)

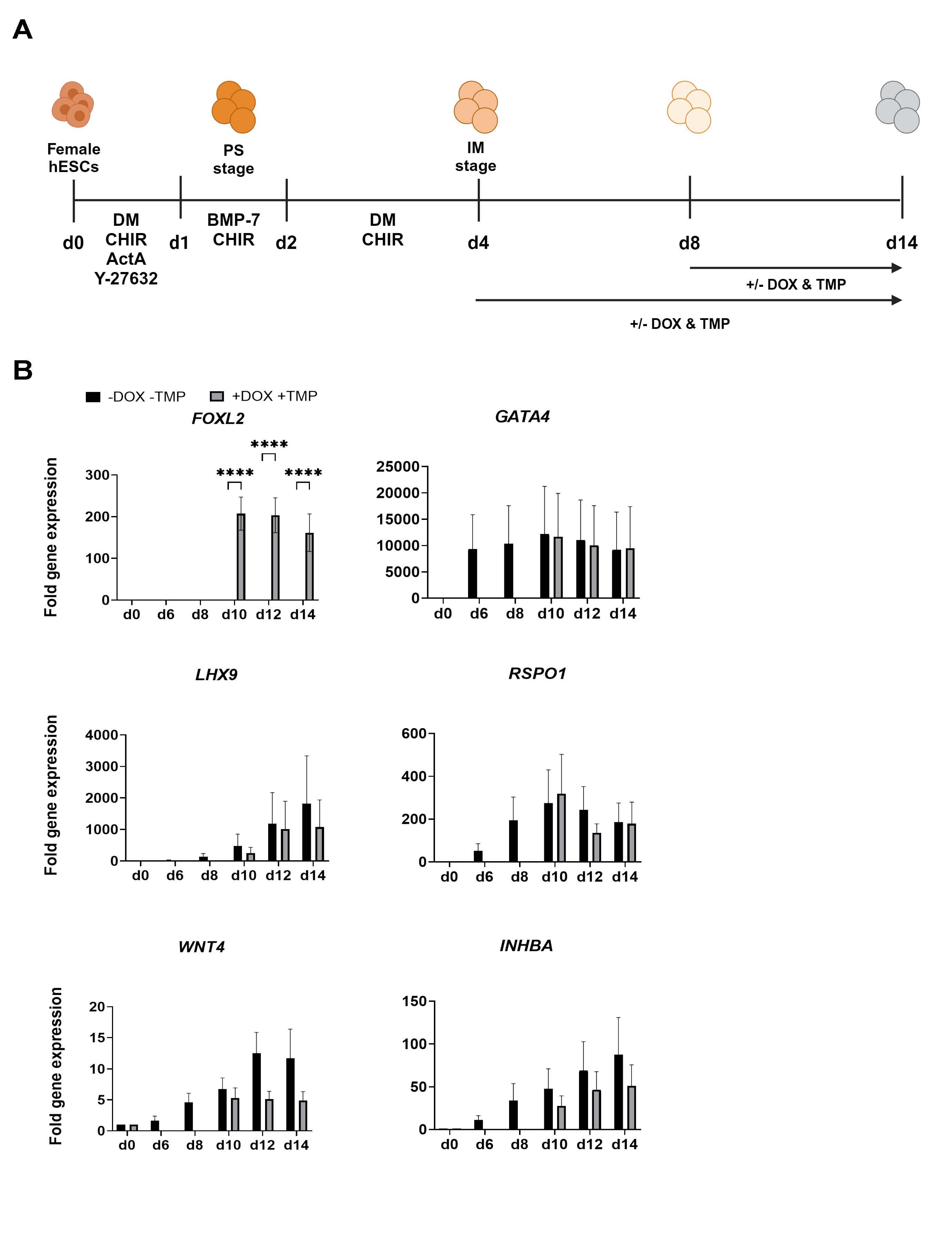

Supplement: Supplementary file 1 — Supplementary Figure 1: FOXL2 induction optimisation showing that day 8 is a sub-optimal induction day. (A) A schematic representation of the 14-day gonadal differentiation protocol including the different small molecules, growth factors, inhibitors used to steer female hESCs towards the IM stage and with the matching developmental stages. Arrows showing the start and end of the FOXL2 induction through addition of the antibiotics DOX and TMP. Created in https://BioRender.com. (B) RT-qPCR analysis of FOXL2 induction at day 8 of gonadal differentiation. FOXL2 was upregulated through the addition of DOX and TMP and minimally downregulated the gonadal markers GATA4, LHX9, RSPO1, WNT4 and INHBA. Data are reported as mean ± SEM, n = 3 biological replicates. The fold change is presented in comparison to d0 (undifferentiated cells) gene expression levels. Two-way ANOVA; 0.1234 (ns), 0.0332 (*), 0.0021 (**), 0.0002 (***), 0.0001 (****). ActA, activin A; BMP, bone morphogenetic protein; CHIR, CHIR-99021; DM, dorsomorphin; hESCs, human embryonic stem cells; IM, intermediate mesoderm; PS, primitive streak; d, day of differentiation; DOX, doxycycline hyclate; TMP, trimethoprim [file 12958_2025_1377_MOESM1_ESM.jpg]

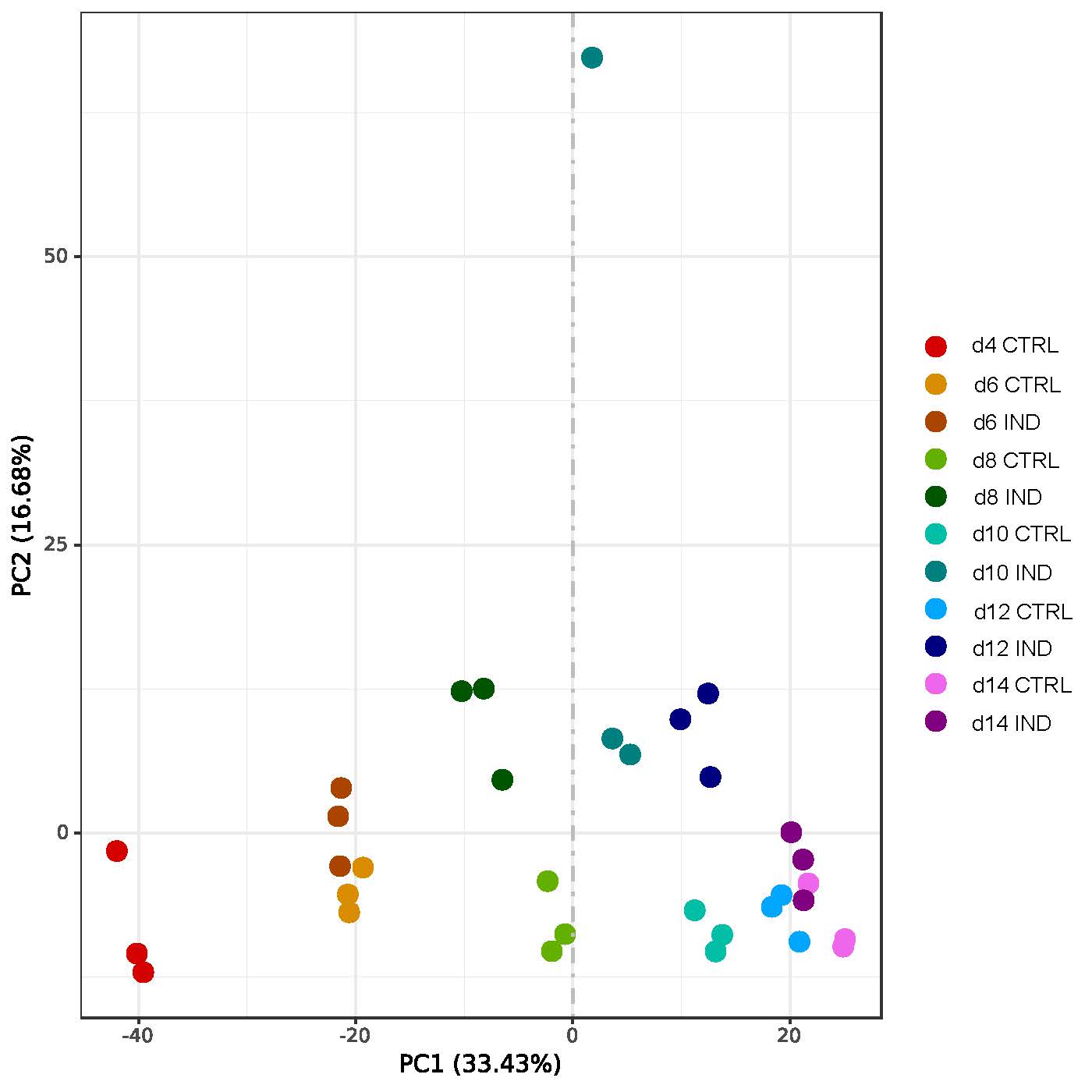

Supplement: Supplementary file 2 — Supplementary Figure 2: Bulk RNA-sequencing showed differences between the control (-DOX-TMP) and induced (+ DOX + TMP) conditions and between the different time points. Principal component analysis showing the divergence between samples from the different timepoints of gonadal differentiation and between control (CTRL) and induced (IND) conditions. Principal component 1 shows the effect of gonadal differentiation and principal component 2 shows the effect of FOXL2 induction. d; day of differentiation; PC, principal component [file 12958_2025_1377_MOESM2_ESM.jpg]

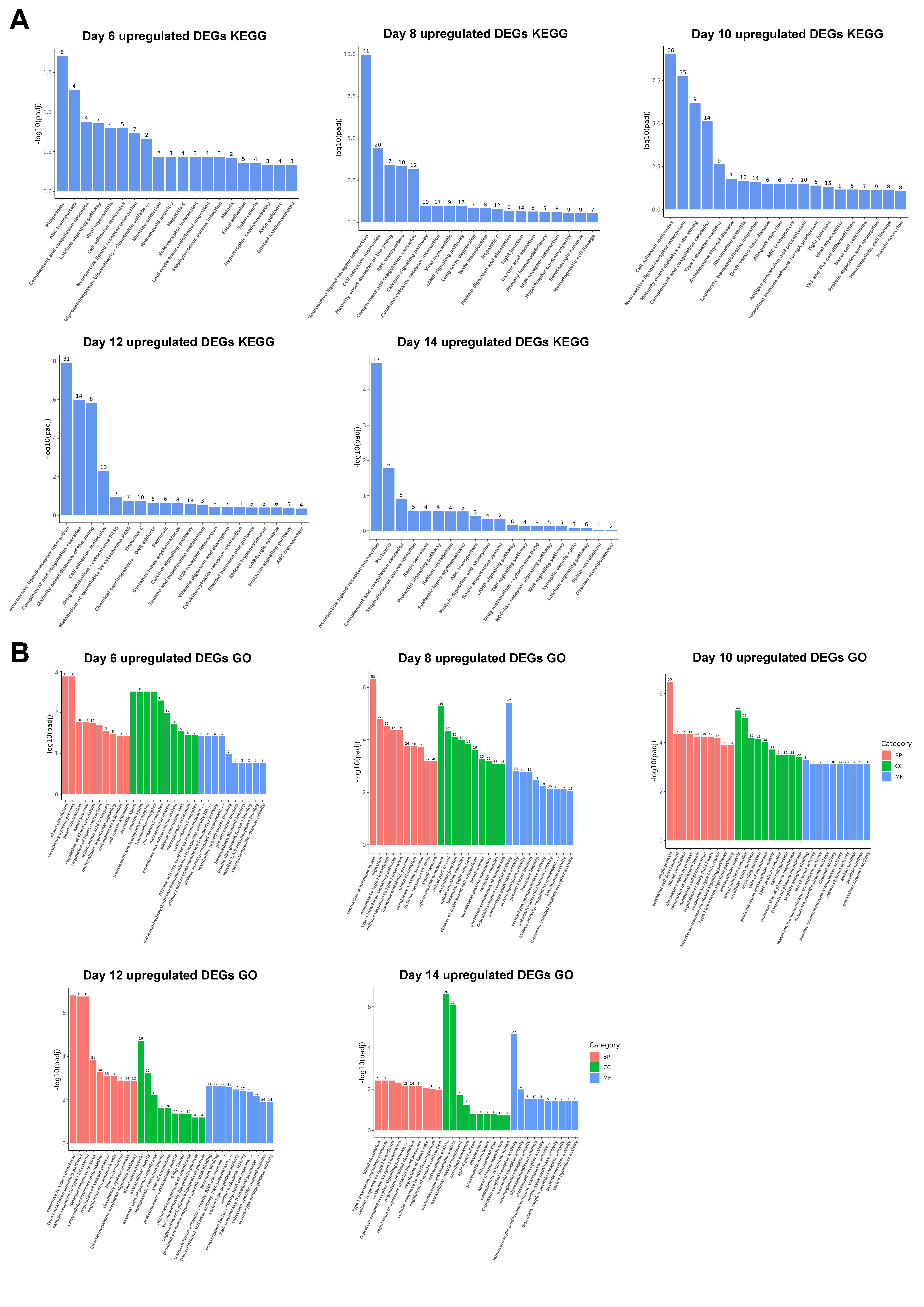

Supplement: Supplementary file 3 — Supplementary Figure 3: FOXL2 induction upregulates pathways associated with cell adhesion, extracellular matrix, and junctions. (A) Bar charts showing Kyoto encyclopaedia of genes and genomes (KEGG) pathways associated with upregulated differentially expressed genes (DEGs) at different time points during the gonadal differentiation. Numbers above bars signify the number of genes associated with the specific pathway. (B) Bar charts showing gene ontology (GO) pathways associated with the upregulated DEGs at different time points during the differentiation. Numbers above the bars signify the number of genes associated with the specific pathway. Bar charts are divided into three categories: biological process (BP), cellular component (CC) and molecular function (MF) [file 12958_2025_1377_MOESM3_ESM.jpg]

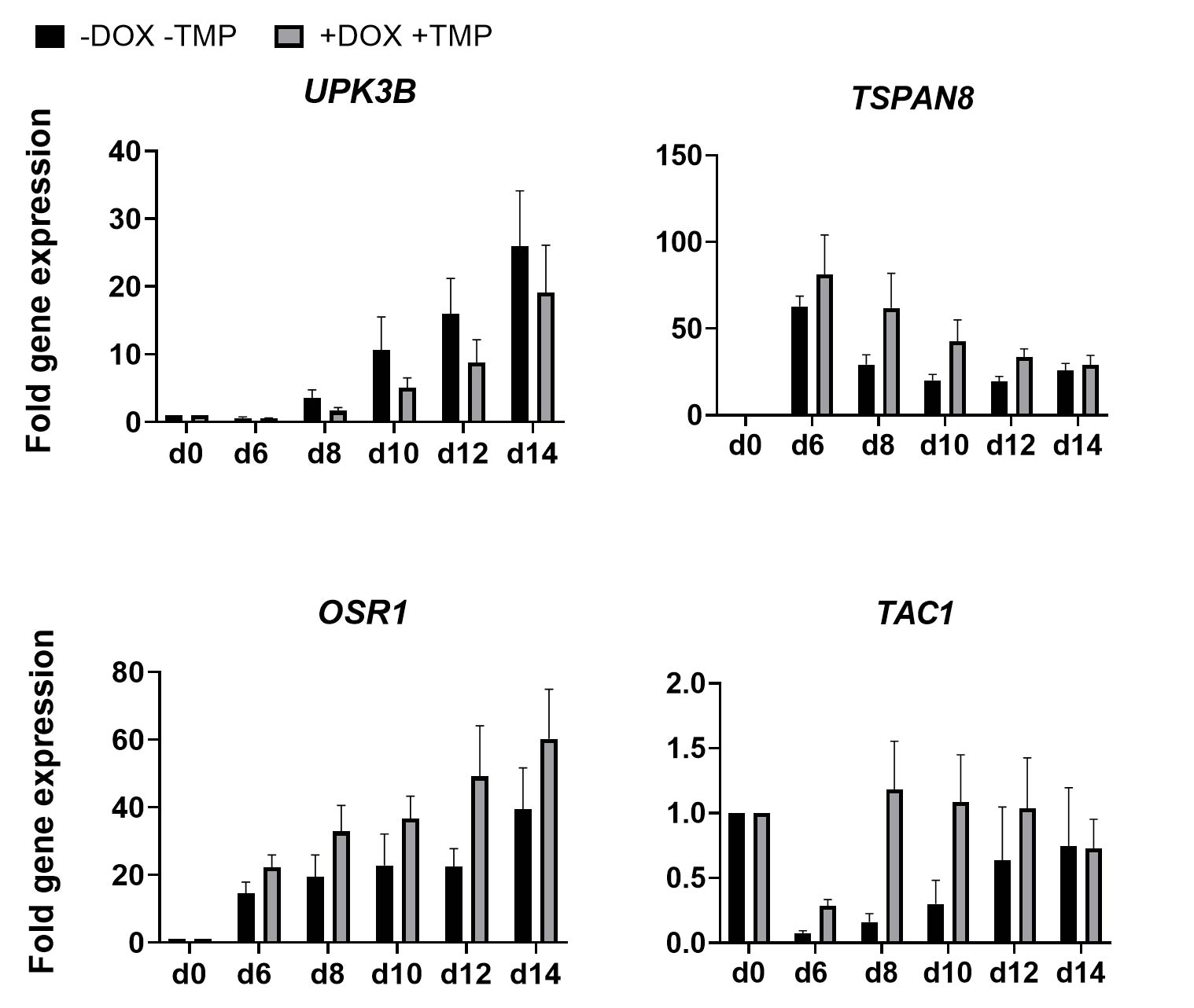

Supplement: Supplementary file 4 — Supplementary Figure 4: RT-qPCR validation confirms bulk RNA-seq results. RT-qPCR analysis shows the downregulation of coelomic epithelial marker UPK3B and the upregulation of ESGC markers TSPAN8, OSR1 and TAC1 upon FOXL2 induction at day 4 of gonadal differentiation. The fold change is presented in comparison to d0 (undifferentiated cells) gene expression levels. Data are reported as mean ± SEM, n = 4 biological replicates. Two-way ANOVA; 0.1234 (ns), 0.0332 (*), 0.0021 (**), 0.0002 (***), 0.0001 (****) [file 12958_2025_1377_MOESM4_ESM.jpg]
